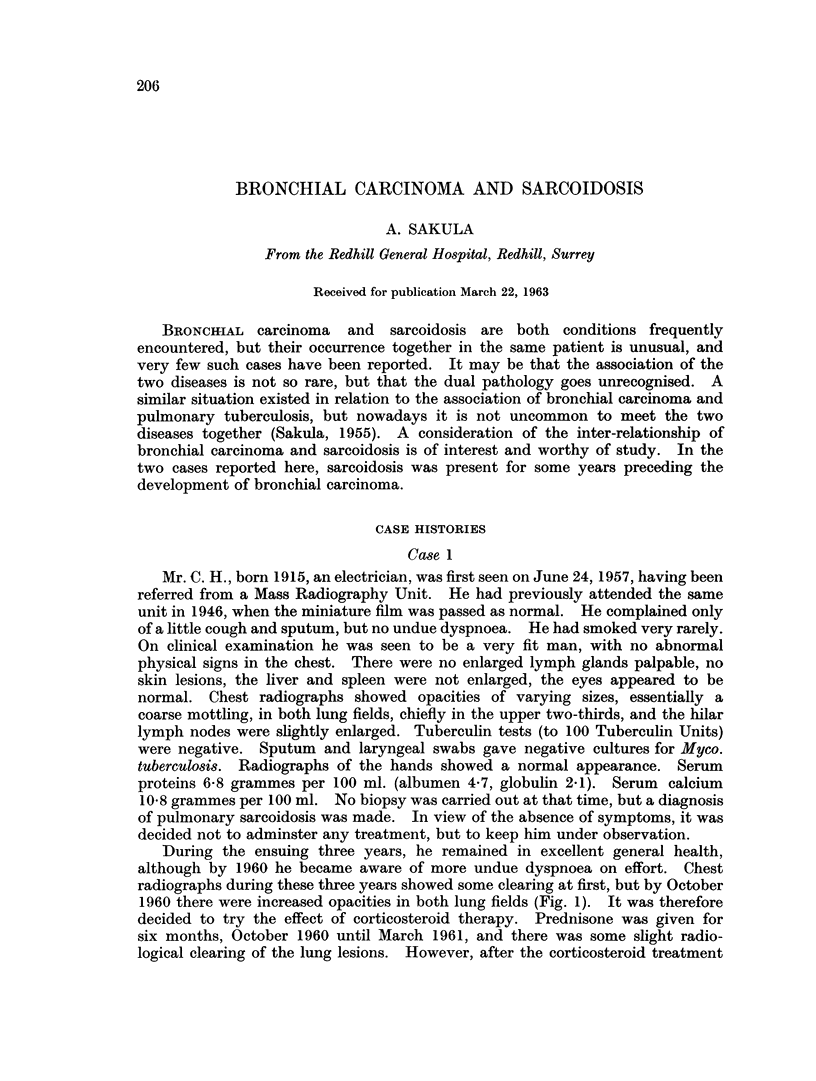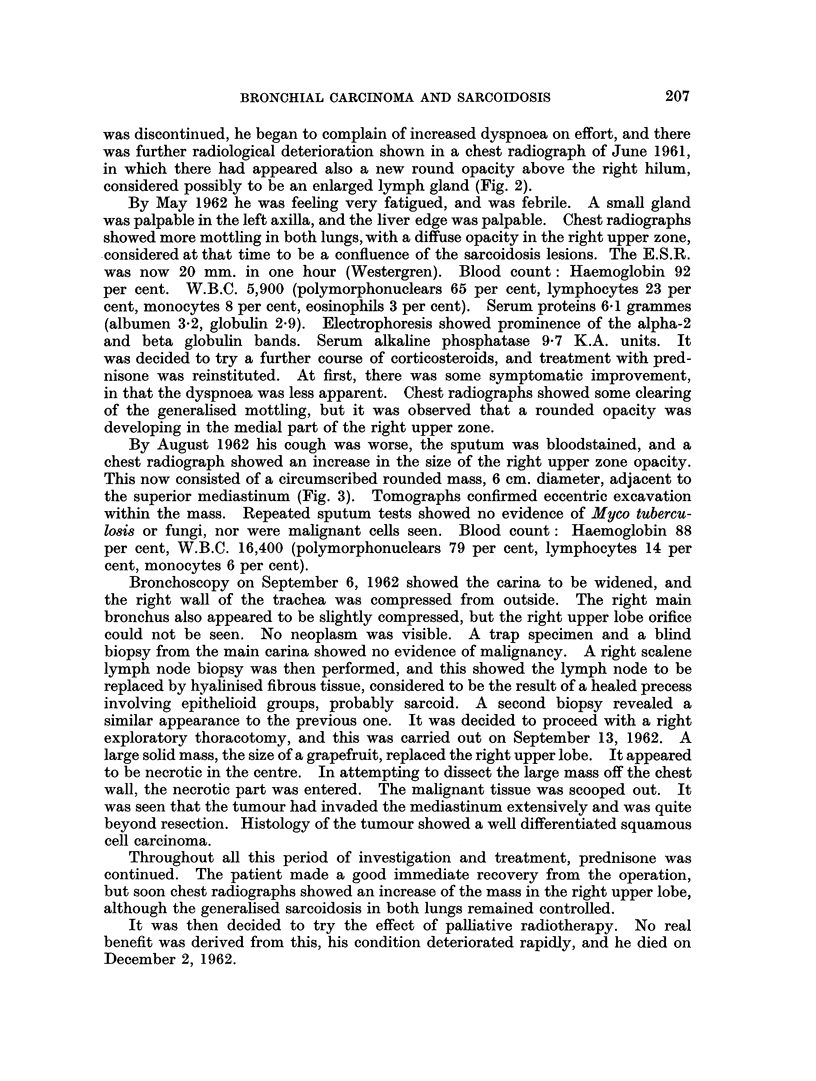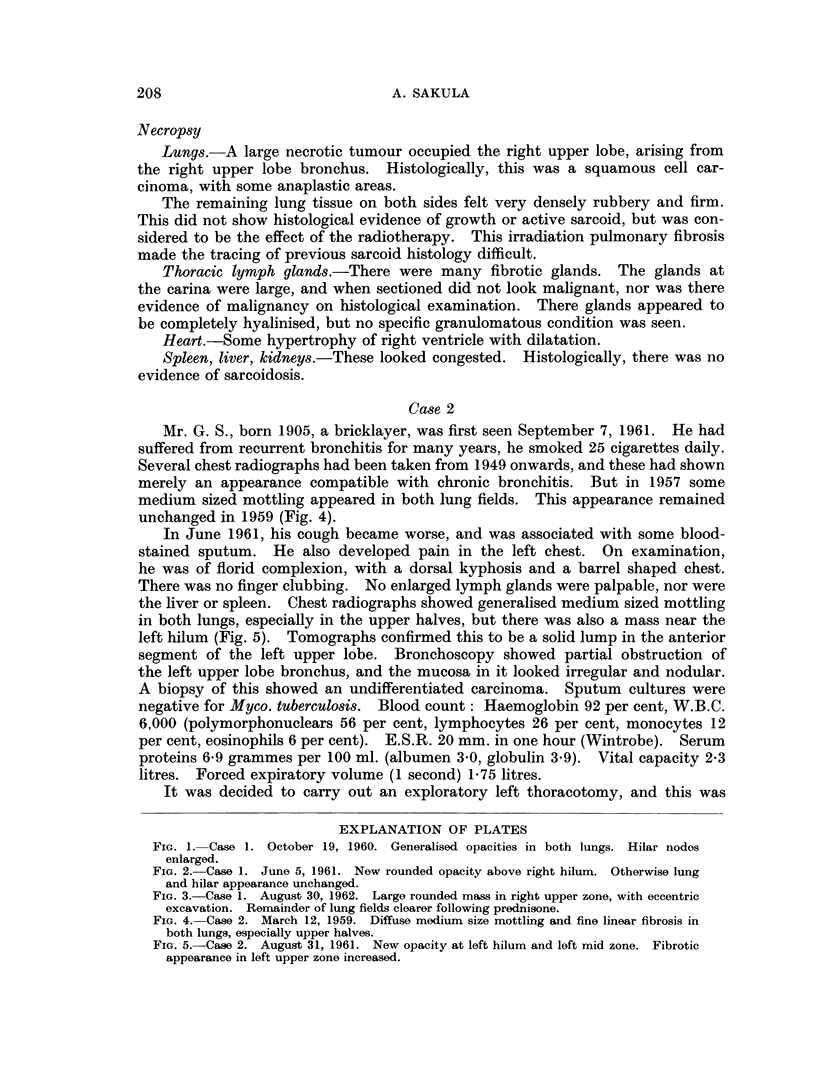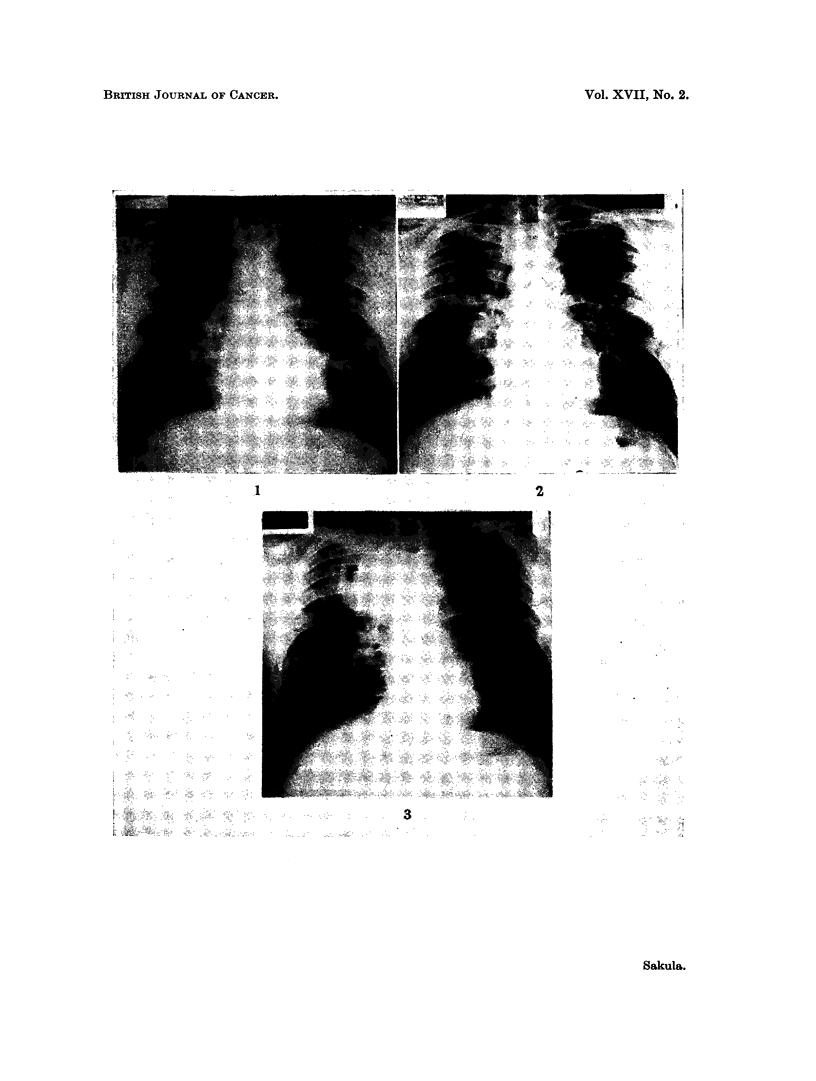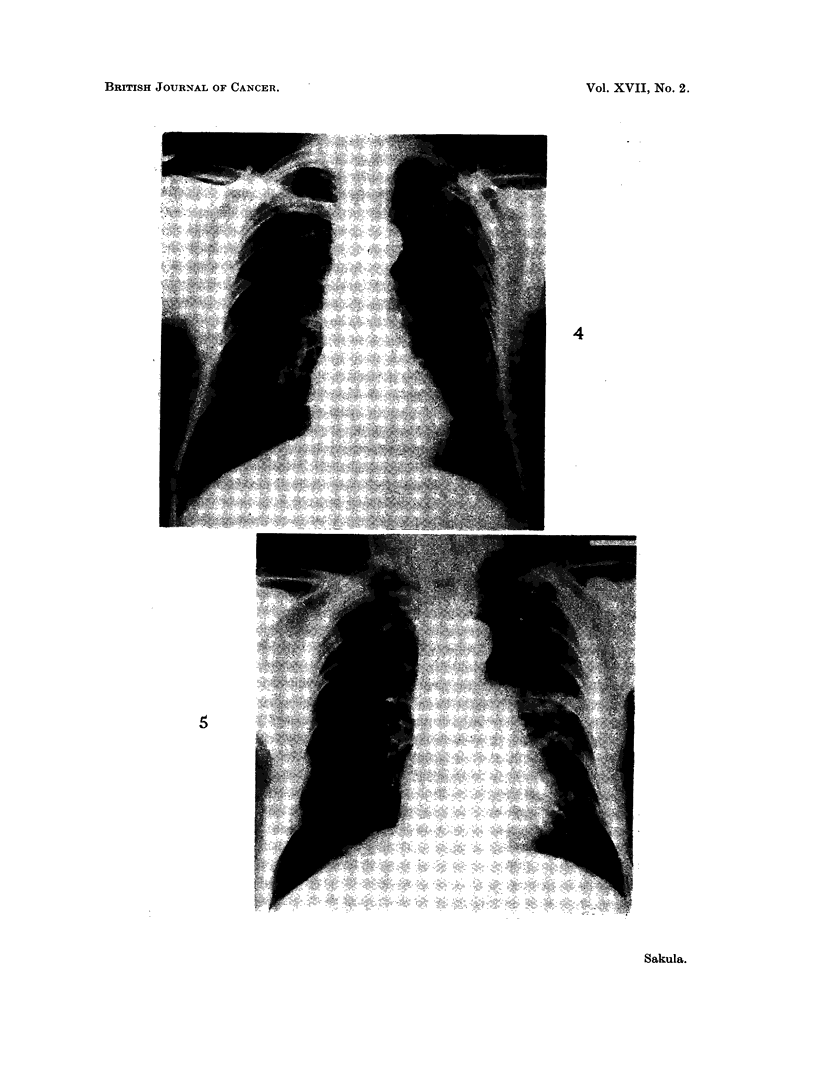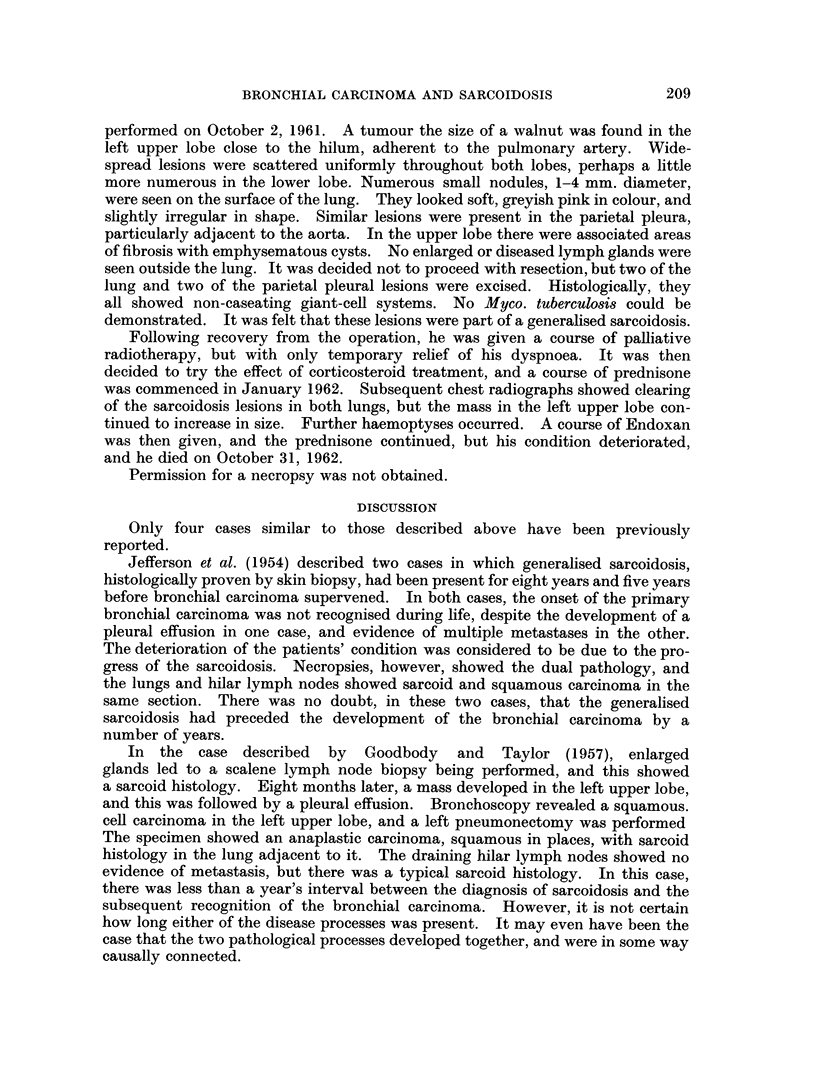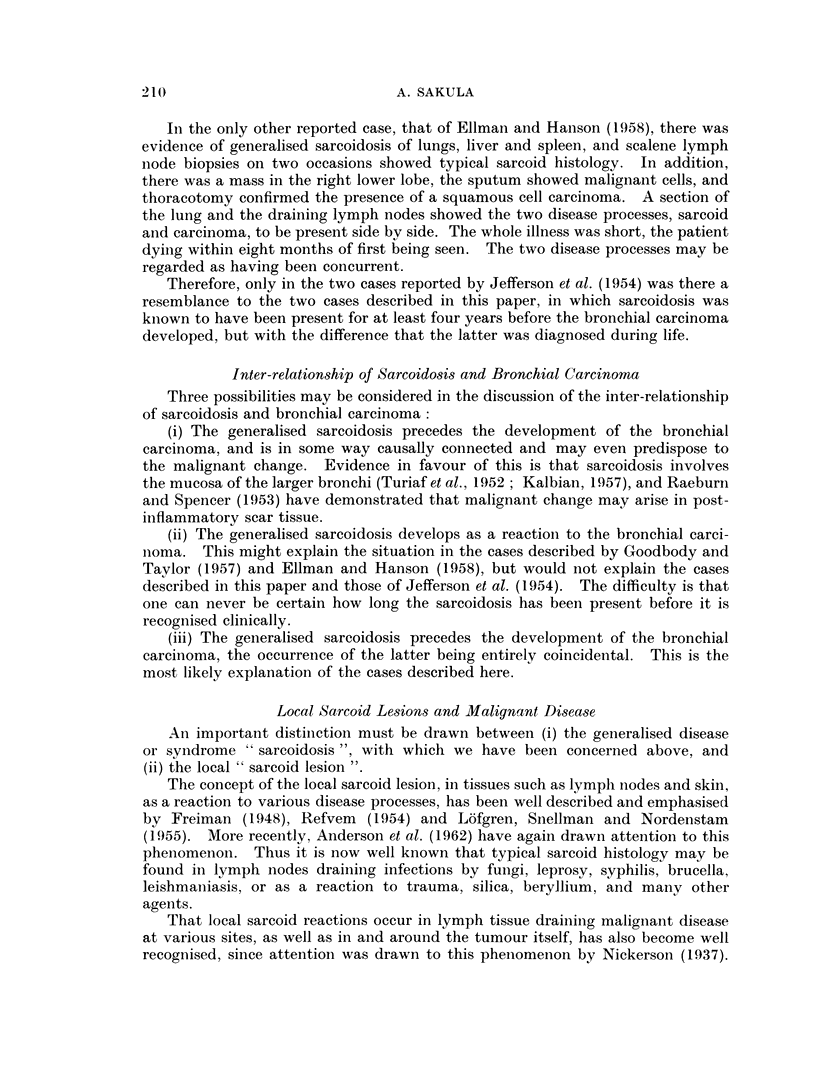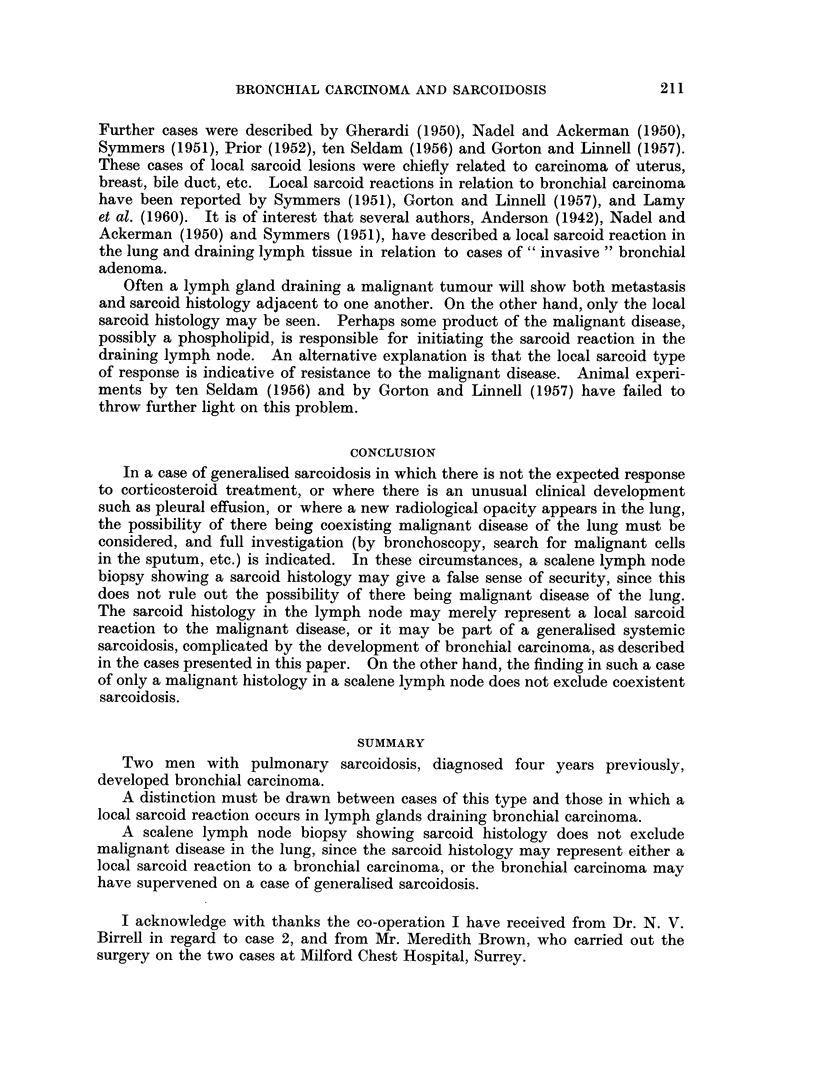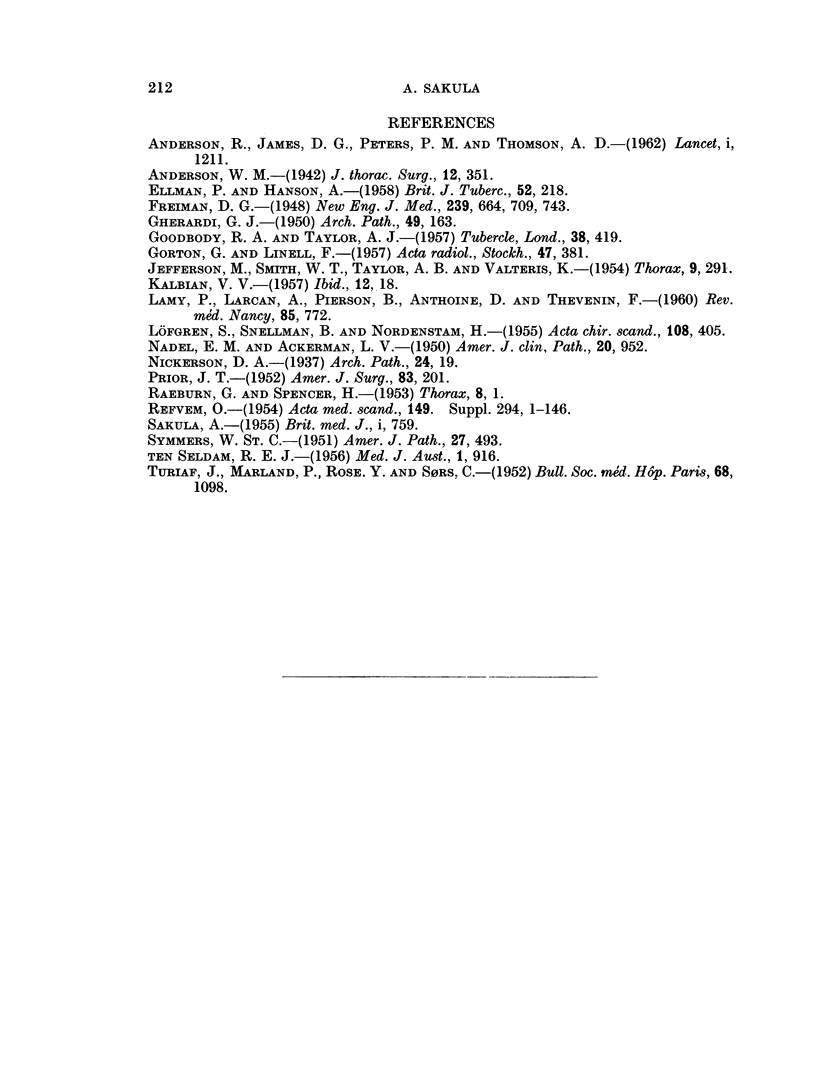# Bronchial Carcinoma and Sarcoidosis

**DOI:** 10.1038/bjc.1963.29

**Published:** 1963-06

**Authors:** A. Sakula

## Abstract

**Images:**


### BRONCHIAL CARCINOMA AND SARCOIDOSIS

A. SAKULA

*From the Redhill General Hospital, Redhill, Surrey*

Received for publication March 22, 1963

BRONCHIAL carcinoma and sarcoidosis are both conditions frequently encountered, but their occurrence together in the same patient is unusual, and very few such cases have been reported. It may be that the association of the two diseases is not so rare, but that the dual pathology goes unrecognised. A similar situation existed in relation to the association of bronchial carcinoma and pulmonary tuberculosis, but nowadays it is not uncommon to meet the two diseases together (Sakula, 1955). A consideration of the inter-relationship of bronchial carcinoma and sarcoidosis is of interest and worthy of study. In the two cases reported here, sarcoidosis was present for some years preceding the development of bronchial carcinoma.

### CASE HISTORIES

### *Case* 1

Mr. C. H., born 1915, an electrician, was first seen on June 24, 1957, having been referred from a Mass Radiography Unit. He had previously attended the same unit in 1946, when the miniature film was passed as normal. He complained only of a little cough and sputum, but no undue dyspnoea. He had smoked very rarely. On clinical examination he was seen to be a very fit man, with no abnormal physical signs in the chest. There were no enlarged lymph glands palpable, no skin lesions, the liver and spleen were not enlarged, the eyes appeared to be normal. Chest radiographs showed opacities of varying sizes, essentially a coarse mottling, in both lung fields, chiefly in the upper two-thirds, and the hilar lymph nodes were slightly enlarged. Tuberculin tests (to 100 Tuberculin Units) were negative. Sputum and laryngeal swabs gave negative cultures for *Myco. tuberculosis*. Radiographs of the hands showed a normal appearance. Serum proteins 6·8 grammes per 100 ml. (albumen 4·7, globulin 2·1). Serum calcium 10·8 grammes per 100 ml. No biopsy was carried out at that time, but a diagnosis of pulmonary sarcoidosis was made. In view of the absence of symptoms, it was decided not to adminster any treatment, but to keep him under observation.

During the ensuing three years, he remained in excellent general health, although by 1960 he became aware of more undue dyspnoea on effort. Chest radiographs during these three years showed some clearing at first, but by October 1960 there were increased opacities in both lung fields (Fig. 1). It was therefore decided to try the effect of corticosteroid therapy. Prednisone was given for six months, October 1960 until March 1961, and there was some slight radiological clearing of the lung lesions. However, after the corticosteroid treatment was discontinued, he began to complain of increased dyspnoea on effort, and there was further radiological deterioration shown in a chest radiograph of June 1961, in which there had appeared also a new round opacity above the right hilum, considered possibly to be an enlarged lymph gland (Fig. 2).

By May 1962 he was feeling very fatigued, and was febrile. A small gland was palpable in the left axilla, and the liver edge was palpable. Chest radiographs showed more mottling in both lungs, with a diffuse opacity in the right upper zone, considered at that time to be a confluence of the sarcoidosis lesions. The E.S.R. was now 20 mm. in one hour (Westergren). Blood count: Haemoglobin 92 per cent. W.B.C. 5,900 (polymorphonuclears 65 per cent, lymphocytes 23 per cent, monocytes 8 per cent, eosinophils 3 per cent). Serum proteins 6·1 grammes (albumen 3·2, globulin 2·9). Electrophoresis showed prominence of the alpha-2 and beta globulin bands. Serum alkaline phosphatase 9·7 K.A. units. It was decided to try a further course of corticosteroids, and treatment with prednisone was reinstituted. At first, there was some symptomatic improvement, in that the dyspnoea was less apparent. Chest radiographs showed some clearing of the generalised mottling, but it was observed that a rounded opacity was developing in the medial part of the right upper zone.

By August 1962 his cough was worse, the sputum was bloodstained, and a chest radiograph showed an increase in the size of the right upper zone opacity. This now consisted of a circumscribed rounded mass, 6 cm. diameter, adjacent to the superior mediastinum (Fig. 3). Tomographs confirmed eccentric excavation within the mass. Repeated sputum tests showed no evidence of *Myco tuberculosis* or fungi, nor were malignant cells seen. Blood count: Haemoglobin 88 per cent, W.B.C. 16,400 (polymorphonuclears 79 per cent, lymphocytes 14 per cent, monocytes 6 per cent).

Bronchoscopy on September 6, 1962 showed the carina to be widened, and the right wall of the trachea was compressed from outside. The right main bronchus also appeared to be slightly compressed, but the right upper lobe orifice could not be seen. No neoplasm was visible. A trap specimen and a blind biopsy from the main carina showed no evidence of malignancy. A right scalene lymph node biopsy was then performed, and this showed the lymph node to be replaced by hyalinised fibrous tissue, considered to be the result of a healed precess involving epithelioid groups, probably sarcoid. A second biopsy revealed a similar appearance to the previous one. It was decided to proceed with a right exploratory thoracotomy, and this was carried out on September 13, 1962. A large solid mass, the size of a grapefruit, replaced the right upper lobe. It appeared to be necrotic in the centre. In attempting to dissect the large mass off the chest wall, the necrotic part was entered. The malignant tissue was scooped out. It was seen that the tumour had invaded the mediastinum extensively and was quite beyond resection. Histology of the tumour showed a well differentiated squamous cell carcinoma.

Throughout all this period of investigation and treatment, prednisone was continued. The patient made a good immediate recovery from the operation, but soon chest radiographs showed an increase of the mass in the right upper lobe, although the generalised sarcoidosis in both lungs remained controlled.

It was then decided to try the effect of palliative radiotherapy. No real benefit was derived from this, his condition deteriorated rapidly, and he died on December 2, 1962.

*Necropsy*

*Lungs.*—A large necrotic tumour occupied the right upper lobe, arising from the right upper lobe bronchus. Histologically, this was a squamous cell carcinoma, with some anaplastic areas.

The remaining lung tissue on both sides felt very densely rubbery and firm. This did not show histological evidence of growth or active sarcoid, but was considered to be the effect of the radiotherapy. This irradiation pulmonary fibrosis made the tracing of previous sarcoid histology difficult.

*Thoracic lymph glands.*—There were many fibrotic glands. The glands at the carina were large, and when sectioned did not look malignant, nor was there evidence of malignancy on histological examination. There glands appeared to be completely hyalinised, but no specific granulomatous condition was seen.

*Heart.*—Some hypertrophy of right ventricle with dilatation.

*Spleen, liver, kidneys.*—These looked congested. Histologically, there was no evidence of sarcoidosis.

*Case* 2

Mr. G. S., born 1905, a bricklayer, was first seen September 7, 1961. He had suffered from recurrent bronchitis for many years, he smoked 25 cigarettes daily. Several chest radiographs had been taken from 1949 onwards, and these had shown merely an appearance compatible with chronic bronchitis. But in 1957 some medium sized mottling appeared in both lung fields. This appearance remained unchanged in 1959 (Fig. 4).

In June 1961, his cough became worse, and was associated with some blood-stained sputum. He also developed pain in the left chest. On examination, he was of florid complexion, with a dorsal kyphosis and a barrel shaped chest. There was no finger clubbing. No enlarged lymph glands were palpable, nor were the liver or spleen. Chest radiographs showed generalised medium sized mottling in both lungs, especially in the upper halves, but there was also a mass near the left hilum (Fig. 5). Tomographs confirmed this to be a solid lump in the anterior segment of the left upper lobe. Bronchoscopy showed partial obstruction of the left upper lobe bronchus, and the mucosa in it looked irregular and nodular. A biopsy of this showed an undifferentiated carcinoma. Sputum cultures were negative for *Myco. tuberculosis.* Blood count : Haemoglobin 92 per cent, W.B.C. 6,000 (polymorphonuclears 56 per cent, lymphocytes 26 per cent, monocytes 12 per cent, eosinophils 6 per cent). E.S.R. 20 mm. in one hour (Wintrobe). Serum proteins 6·9 grammes per 100 ml. (albumen 3·0, globulin 3·9). Vital capacity 2·3 litres. Forced expiratory volume (1 second) 1·75 litres.

It was decided to carry out an exploratory left thoracotomy, and this was

---

EXPLANATION OF PLATES

FIG. 1.—Case 1. October 19, 1960. Generalised opacities in both lungs. Hilar nodes enlarged.

FIG. 2.—Case 1. June 5, 1961. New rounded opacity above right hilum. Otherwise lung and hilar appearance unchanged.

FIG. 3.—Case 1. August 30, 1962. Large rounded mass in right upper zone, with eccentric excavation. Remainder of lung fields clearer following prednisone.

FIG. 4.—Case 2. March 12, 1959. Diffuse medium size mottling and fine linear fibrosis in both lungs, especially upper halves.

FIG. 5.—Case 2. August 31, 1961. New opacity at left hilum and left mid zone. Fibrotic appearance in left upper zone increased.

1

2

3

Sakula.

4

5

Sakula.

performed on October 2, 1961. A tumour the size of a walnut was found in the left upper lobe close to the hilum, adherent to the pulmonary artery. Widespread lesions were scattered uniformly throughout both lobes, perhaps a little more numerous in the lower lobe. Numerous small nodules, 1–4 mm. diameter, were seen on the surface of the lung. They looked soft, greyish pink in colour, and slightly irregular in shape. Similar lesions were present in the parietal pleura, particularly adjacent to the aorta. In the upper lobe there were associated areas of fibrosis with emphysematous cysts. No enlarged or diseased lymph glands were seen outside the lung. It was decided not to proceed with resection, but two of the lung and two of the parietal pleural lesions were excised. Histologically, they all showed non-caseating giant-cell systems. No *Myco. tuberculosis* could be demonstrated. It was felt that these lesions were part of a generalised sarcoidosis.

Following recovery from the operation, he was given a course of palliative radiotherapy, but with only temporary relief of his dyspnoea. It was then decided to try the effect of corticosteroid treatment, and a course of prednisone was commenced in January 1962. Subsequent chest radiographs showed clearing of the sarcoidosis lesions in both lungs, but the mass in the left upper lobe continued to increase in size. Further haemoptyses occurred. A course of Endoxan was then given, and the prednisone continued, but his condition deteriorated, and he died on October 31, 1962.

Permission for a necropsy was not obtained.

### DISCUSSION

Only four cases similar to those described above have been previously reported.

Jefferson *et al.* (1954) described two cases in which generalised sarcoidosis, histologically proven by skin biopsy, had been present for eight years and five years before bronchial carcinoma supervened. In both cases, the onset of the primary bronchial carcinoma was not recognised during life, despite the development of a pleural effusion in one case, and evidence of multiple metastases in the other. The deterioration of the patients' condition was considered to be due to the progress of the sarcoidosis. Necropsies, however, showed the dual pathology, and the lungs and hilar lymph nodes showed sarcoid and squamous carcinoma in the same section. There was no doubt, in these two cases, that the generalised sarcoidosis had preceded the development of the bronchial carcinoma by a number of years.

In the case described by Goodbody and Taylor (1957), enlarged glands led to a scalene lymph node biopsy being performed, and this showed a sarcoid histology. Eight months later, a mass developed in the left upper lobe, and this was followed by a pleural effusion. Bronchoscopy revealed a squamous. cell carcinoma in the left upper lobe, and a left pneumonectomy was performed The specimen showed an anaplastic carcinoma, squamous in places, with sarcoid histology in the lung adjacent to it. The draining hilar lymph nodes showed no evidence of metastasis, but there was a typical sarcoid histology. In this case, there was less than a year's interval between the diagnosis of sarcoidosis and the subsequent recognition of the bronchial carcinoma. However, it is not certain how long either of the disease processes was present. It may even have been the case that the two pathological processes developed together, and were in some way causally connected.

In the only other reported case, that of Ellman and Hanson (1958), there was evidence of generalised sarcoidosis of lungs, liver and spleen, and scalene lymph node biopsies on two occasions showed typical sarcoid histology. In addition, there was a mass in the right lower lobe, the sputum showed malignant cells, and thoracotomy confirmed the presence of a squamous cell carcinoma. A section of the lung and the draining lymph nodes showed the two disease processes, sarcoid and carcinoma, to be present side by side. The whole illness was short, the patient dying within eight months of first being seen. The two disease processes may be regarded as having been concurrent.

Therefore, only in the two cases reported by Jefferson *et al.* (1954) was there a resemblance to the two cases described in this paper, in which sarcoidosis was known to have been present for at least four years before the bronchial carcinoma developed, but with the difference that the latter was diagnosed during life.

### *Inter-relationship of Sarcoidosis and Bronchial Carcinoma*

Three possibilities may be considered in the discussion of the inter-relationship of sarcoidosis and bronchial carcinoma :

(i) The generalised sarcoidosis precedes the development of the bronchial carcinoma, and is in some way causally connected and may even predispose to the malignant change. Evidence in favour of this is that sarcoidosis involves the mucosa of the larger bronchi (Turiaf *et al.*, 1952 ; Kalbian, 1957), and Raeburn and Spencer (1953) have demonstrated that malignant change may arise in post-inflammatory scar tissue.

(ii) The generalised sarcoidosis develops as a reaction to the bronchial carcinoma. This might explain the situation in the cases described by Goodbody and Taylor (1957) and Ellman and Hanson (1958), but would not explain the cases described in this paper and those of Jefferson *et al.* (1954). The difficulty is that one can never be certain how long the sarcoidosis has been present before it is recognised clinically.

(iii) The generalised sarcoidosis precedes the development of the bronchial carcinoma, the occurrence of the latter being entirely coincidental. This is the most likely explanation of the cases described here.

### *Local Sarcoid Lesions and Malignant Disease*

An important distinction must be drawn between (i) the generalised disease or syndrome " sarcoidosis ", with which we have been concerned above, and (ii) the local " sarcoid lesion ".

The concept of the local sarcoid lesion, in tissues such as lymph nodes and skin, as a reaction to various disease processes, has been well described and emphasised by Freiman (1948), Refvem (1954) and Löfgren, Snellman and Nordenstam (1955). More recently, Anderson *et al.* (1962) have again drawn attention to this phenomenon. Thus it is now well known that typical sarcoid histology may be found in lymph nodes draining infections by fungi, leprosy, syphilis, brucella, leishmaniasis, or as a reaction to trauma, silica, beryllium, and many other agents.

That local sarcoid reactions occur in lymph tissue draining malignant disease at various sites, as well as in and around the tumour itself, has also become well recognised, since attention was drawn to this phenomenon by Nickerson (1937).

Further cases were described by Gherardi (1950), Nadel and Ackerman (1950), Symmers (1951), Prior (1952), ten Seldam (1956) and Gorton and Linnell (1957). These cases of local sarcoid lesions were chiefly related to carcinoma of uterus, breast, bile duct, etc. Local sarcoid reactions in relation to bronchial carcinoma have been reported by Symmers (1951), Gorton and Linnell (1957), and Lamy *et al.* (1960). It is of interest that several authors, Anderson (1942), Nadel and Ackerman (1950) and Symmers (1951), have described a local sarcoid reaction in the lung and draining lymph tissue in relation to cases of " invasive " bronchial adenoma.

Often a lymph gland draining a malignant tumour will show both metastasis and sarcoid histology adjacent to one another. On the other hand, only the local sarcoid histology may be seen. Perhaps some product of the malignant disease, possibly a phospholipid, is responsible for initiating the sarcoid reaction in the draining lymph node. An alternative explanation is that the local sarcoid type of response is indicative of resistance to the malignant disease. Animal experiments by ten Seldam (1956) and by Gorton and Linnell (1957) have failed to throw further light on this problem.

### CONCLUSION

In a case of generalised sarcoidosis in which there is not the expected response to corticosteroid treatment, or where there is an unusual clinical development such as pleural effusion, or where a new radiological opacity appears in the lung, the possibility of there being coexisting malignant disease of the lung must be considered, and full investigation (by bronchoscopy, search for malignant cells in the sputum, etc.) is indicated. In these circumstances, a scalene lymph node biopsy showing a sarcoid histology may give a false sense of security, since this does not rule out the possibility of there being malignant disease of the lung. The sarcoid histology in the lymph node may merely represent a local sarcoid reaction to the malignant disease, or it may be part of a generalised systemic sarcoidosis, complicated by the development of bronchial carcinoma, as described in the cases presented in this paper. On the other hand, the finding in such a case of only a malignant histology in a scalene lymph node does not exclude coexistent sarcoidosis.

### SUMMARY

Two men with pulmonary sarcoidosis, diagnosed four years previously, developed bronchial carcinoma.

A distinction must be drawn between cases of this type and those in which a local sarcoid reaction occurs in lymph glands draining bronchial carcinoma.

A scalene lymph node biopsy showing sarcoid histology does not exclude malignant disease in the lung, since the sarcoid histology may represent either a local sarcoid reaction to a bronchial carcinoma, or the bronchial carcinoma may have supervened on a case of generalised sarcoidosis.

I acknowledge with thanks the co-operation I have received from Dr. N. V. Birrell in regard to case 2, and from Mr. Meredith Brown, who carried out the surgery on the two cases at Milford Chest Hospital, Surrey.

### REFERENCES

ANDERSON, R., JAMES, D. G., PETERS, P. M. AND THOMSON, A. D.—(1962) *Lancet*, i, 1211.
ANDERSON, W. M.—(1942) *J. thorac. Surg.*, **12**, 351.
ELLMAN, P. AND HANSON, A.—(1958) *Brit. J. Tuberc.*, **52**, 218.
FREIMAN, D. G.—(1948) *New Eng. J. Med.*, **239**, 664, 709, 743.
GHERARDI, G. J.—(1950) *Arch. Path.*, **49**, 163.
GOODBODY, R. A. AND TAYLOR, A. J.—(1957) *Tubercle, Lond.*, **38**, 419.
GORTON, G. AND LINELL, F.—(1957) *Acta radiol., Stockh.*, **47**, 381.
JEFFERSON, M., SMITH, W. T., TAYLOR, A. B. AND VALTERIS, K.—(1954) *Thorax*, **9**, 291.
KALBIAN, V. V.—(1957) *Ibid.*, **12**, 18.
LAMY, P., LARCAN, A., PIERSON, B., ANTHOINE, D. AND THEVENIN, F.—(1960) *Rev. méd. Nancy*, **85**, 772.
LÖFGREN, S., SNELLMAN, B. AND NORDENSTAM, H.—(1955) *Acta chir. scand.*, **108**, 405.
NADEL, E. M. AND ACKERMAN, L. V.—(1950) *Amer. J. clin, Path.*, **20**, 952.
NICKERSON, D. A.—(1937) *Arch. Path.*, **24**, 19.
PRIOR, J. T.—(1952) *Amer. J. Surg.*, **83**, 201.
RAEBURN, G. AND SPENCER, H.—(1953) *Thorax*, **8**, 1.
REFVEM, O.—(1954) *Acta med. scand.*, **149**. Suppl. 294, 1–146.
SAKULA, A.—(1955) *Brit. med. J.*, i, 759.
SYMMERS, W. ST. C.—(1951) *Amer. J. Path.*, **27**, 493.
TEN SELDAM, R. E. J.—(1956) *Med. J. Aust.*, **1**, 916.
TURIAF, J., MARLAND, P., ROSE. Y. AND SØRS, C.—(1952) *Bull. Soc. méd. Hôp. Paris*, **68**, 1098.